# Harnessing Finger Millet to Combat Calcium Deficiency in Humans: Challenges and Prospects

**DOI:** 10.3389/fpls.2017.01311

**Published:** 2017-07-26

**Authors:** Swati Puranik, Jason Kam, Pranav P. Sahu, Rama Yadav, Rakesh K. Srivastava, Henry Ojulong, Rattan Yadav

**Affiliations:** ^1^Institute of Biological, Environmental and Rural Sciences, Aberystwyth University Aberystwyth, United Kingdom; ^2^International Crops Research Institute for the Semi-Arid Tropics Patancheru, India; ^3^International Crops Research Institute for the Semi-Arid Tropics Nairobi, Kenya

**Keywords:** finger millet, calcium, osteoporosis, bioavailability, food processing, biofortification, genetic improvement, plant breeding

## Abstract

Humans require more than 20 mineral elements for healthy body function. Calcium (Ca), one of the essential macromineral, is required in relatively large quantities in the diet for maintaining a sound overall health. Young children, pregnant and nursing women in marginalized and poorest regions of the world, are at highest risk of Ca malnutrition. Elderly population is another group of people most commonly affected by Ca deficiency mainly in the form of osteoporosis and osteopenia. Improved dietary intake of Ca may be the most cost-effective way to meet such deficiencies. Finger millet [*Eleusine coracana* (L.) Gaertn.], a crop with inherently higher Ca content in its grain, is an excellent candidate for understanding genetic mechanisms associated with Ca accumulation in grain crops. Such knowledge will also contribute toward increasing Ca contents in other staple crops consumed on daily basis using plant-breeding (also known as biofortification) methods. However, developing Ca-biofortified finger millet to reach nutritional acceptability faces various challenges. These include identifying and translating the high grain Ca content to an adequately bioavailable form so as to have a positive impact on Ca malnutrition. In this review, we assess some recent advancements and challenges for enrichment of its Ca value and present possible inter-disciplinary prospects for advancing the actual impact of Ca-biofortified finger millet.

## Importance of calcium in human diet

Calcium (Ca) is the fifth most abundant element present in the human body, accounting for up to 1.9% of the body weight in adults (Nordin, 1976). Its main functions are to provide rigidity and structure, mediating vascular and muscular contractions or dilations and nerve signal transmission (Institute of Medicine (US) Standing Committee on the Scientific Evaluation of Dietary Reference Intakes, 1997; Nordin, 1997). Ca may also serve in the protective role against various types of cancer viz. colorectal (Institute of Medicine (US) Committee to Review Dietary Reference Intakes for Vitamin D and Calcium, 2011), ovarian (Goodman et al., 2002), breast (Lin et al., 2007), and prostate (Gao et al., 2005). Although not supported by clinical trials, observational studies have associated higher Ca intakes to lower body weight and reduced adiposity, which may be due to lower intracellular Ca in fat cells leading to a higher fat breakdown (Parikh and Yanovski, 2003). Thus, it may reduce the risk of cardiovascular diseases by lowering intestinal lipid absorption, promoting lipid excretion and decreasing cholesterol concentrations in the blood (Institute of Medicine (US) Committee to Review Dietary Reference Intakes for Vitamin D and Calcium, 2011). Ca is also known to be important for those who have diabetes (Levy et al., 1994; Vestergaard, 2006; Pittas et al., 2007), particularly type 1 diabetics, who in general have lower bone mineral density than healthy subjects (Ma et al., 2012; Oei et al., 2013).

Given its importance, authorities like the Food and Agriculture Organization (FAO) of the United Nations have set up a recommended daily intake (RDI) of Ca based on age, life stage and gender (Food Agricultural Organization of the United Nations, 2002). During the phases of active growth, Ca equilibrium in the body maintains a stable bone mass. Therefore, FAO recommends that children of 1–3 years consume 500 mg Ca/day, 4–6 years consume 600 mg Ca/day and 7–9 years consume 700 mg Ca/day, which should be increased to 1,300 mg/day during 10–18 years (Food Agricultural Organization of the United Nations, 2002). About 1,000 mg Ca/day is recommended between the ages of 19–65 years in males. The organization also advocates that women should take 1,000 mg Ca each day from 19 years onwards until menopause raising it to 1,200 mg during the last trimester of pregnancy, and to 1,300 mg from 65 years and above (Food Agricultural Organization of the United Nations, 2002).

However, after 50 years in men and in menopausal women, the onset of bone decalcification and demineralization leads to reductions in bone mass causing the disease osteoporosis (Michaelsson et al., 2005). According to International Osteoporosis Foundation, it is a significant problem both in the developed as well as in developing nations (http://www.iofbonehealth.org/facts-statistics). The World Health Organization (WHO) has declared osteoporosis as the next main public healthcare concern globally, after cardiovascular diseases (CVDs), inflicting almost 75 million people in Europe, the United States of America and Japan alone (Consensus Development Statement, 1997; Haldipur, 2003). With a growing elderly population, osteoporosis is threatening to become a major global economic burden for the already stretched healthcare system across the globe. By 2050, the worldwide cost of treating osteoporosis is forecasted to USD 131.5 billion (Lindsay et al., 2001). Thus, in order to prevent Ca deficiency at this age, it is necessary to sustain the recommended daily intake (RDI) of 1,300 mg/day (especially after 65 years; Food Agricultural Organization of the United Nations, 2002) to ensure maximal bone mass during the developmental stages and to have reduced bone mass loss during old age (Michaelsson, 2009).

Despite the importance of adequate Ca intake for human health and wellbeing, the WHO estimates that low dietary intake of Ca is common across the world (WHO, 2006). On a global scale, it presents a large risk which gets graver in underdeveloped regions of the world. However, the absence of reliable and practical indicators in these areas provides insufficient data, which is a challenge for resolving the actual global status for the prevalence of Ca deficiency. It has recently been determined (mainly based on food supply) that 3.5 billion people were at the risk of Ca deficiency in 2011, with approximately 90% of the affected individuals in Africa and Asia (Kumssa et al., 2015). Most of these regions have an agriculture-based economy and large segments of these populations are typically dependent on what they grow and produce for their Ca need. In such situations, staple crops that can offer adequate Ca requirements, especially for people of low income groups in these countries, are highly recommended. One such Ca-rich, traditional and locally well-adapted crop is finger millet. As opposed to nutritionally deficient cereals, such as rice, its regular consumption has a vast potential to curb the incidences of Ca deficiency. Finger millet also possesses many other health-benefitting traits. For example, it has been highlighted that as a model nutraceutical crop, finger millet can provide excellent solutions to food and health security issues (Kumar et al., 2016a). Being a stress resilient crop requiring minimal inputs for growth it is especially suited for sustainable agriculture (Gupta et al., 2017). Despite this, it has received very little scientific attention, relative to other crops, such as rice, wheat and maize. Some very recent reviews have provided a comprehensive account of molecular mechanisms that may be involved in calcium nutrition in finger millet and how various approaches, including molecular breeding, functional genomics and transgenic technology can elevate Ca accumulation in its grains (Ganapathy, 2017; Sharma et al., 2017). However, beyond this, in order to advance biofortification of finger millet, challenges associated with its impact on human health also needs to be critically evaluated. With this as focus, we have not only attempted to highlight its potential but also the prospects of advancing the bioaccessibility of grain Ca content even further.

## Strategies to prevent calcium deficiency

Calcium (Ca) deficiency is almost physically undetectable and difficult to diagnose during preliminary stages (Wang et al., 2013), however, if detected, it is usually easy to treat by increasing dietary Ca intake or absorption. Currently, there are many strategies that can be undertaken to manage the problem of Ca deficiency. These mainly include diversification of diet, food fortification, external supplementation and crop biofortification.

Dietary changes by including foods that are naturally rich in Ca, like dairy products, seems to be the most efficient way to prevent Ca deficiency. However, it is not easy to persuade people to go for diversified diets and certain foods cannot be included in the diets. For example, 65% of the world's population is lactose-intolerant and therefore they cannot rely on dairy products for their Ca requirement. Incidentally, most of these lactose-intolerant people live in Asian and African regions (Curry, 2013), which are predominantly dependent on agriculture-based economies. Additionally, due to resource constrain, they may not be able to afford livestock or may solely raise cattle for supplementing income and deny themselves of milk and milk products. Therefore, many communities in these regions, including vegetarians and vegans, need alternative sources to meet their Ca needs.

Another strategy can be an industry-based fortification of foods consumed by target groups. For example, policy decisions in the western world have supported fortification of breakfast cereals, fruit juices, flours and sports drinks with Ca. However, fortification may alter flavor, bioavailability, shelf life or safety of the product, thus making it unpalatable to the consumers. Further, consumers in under-developed nations, with limited education and economic resources, often do not always have access to such fortified foods.

Supplementation is an alternative in which Ca tablets are used to prevent Ca deficiency. Although more accessible to people, this strategy suffers from major drawbacks, mostly associated with their side-effects (Institute of Medicine (US) Committee to Review Dietary Reference Intakes for Vitamin D and Calcium, 2011). The most common content of supplementation pills are the inorganic forms: calcium carbonate and calcium citrate. Large amounts of Ca supplements cause excessive Ca accumulation in vascular and soft tissues like arteries or organs, such as kidneys which can lead to heart attack or kidney stones, respectively (Bolland et al., 2010; Institute of Medicine (US) Committee to Review Dietary Reference Intakes for Vitamin D and Calcium, 2011). Some studies have also linked supplement intake with higher risk of breast cancer, prostate cancer and cardiovascular diseases (Institute of Medicine (US) Committee to Review Dietary Reference Intakes for Vitamin D and Calcium, 2011). Further, taking Ca supplements with meals may also reduce absorption of other minerals like zinc and iron (Cook et al., 1991). Therefore, even though this strategy can potentially reach many people, the associated medical problems make it a non-viable long-term option.

To combat Ca deficiency for a wider and more sustainable impact, alternative solutions that are cost-effective and can easily be adopted especially in the developing countries settings need to be pursued. Genetic biofortification is one such very powerful plant breeding or transgenics-based strategy which can combat the global challenge of micronutrient deficiency (www.copenhagenconsensus.com). As it involves the already established agricultural systems to grow, breed and distribute nutrient-dense staple crops, it can be the most economically and socially feasible approach to integrate nutrition into the diets of the impoverished. Biofortified staple crops have been quantified to have high potential benefits and cost effectiveness as their cost per disability-adjusted life years (DALYs) saved is less than the national per capita income, when compared to other interventions, such as fortification and supplementation (Stein et al., 2005; Asare-Marfo et al., 2014).

Over the last decade several projects, such as those initiated by Harvest Plus, have released nutritionally enhanced rice and wheat varieties (Bouis et al., 2011). Recently, efforts have also commenced for biofortification breeding of small cereals, such as millets. Miles ahead of widely consumed rice and wheat, this group of hardy cereals contribute up to 75% of the total calorie intake for poorest of the poor (O'Kennedy et al., 2006). Therefore, millets hold stronger promise to provide economic, food and nutritional security to people surviving on marginal and resource-poor soils (Muthamilarasan et al., 2016). In this respect, pearl millet has become a target for biofortification of iron and zinc (Manwaring et al., 2016) and has entered the spotlight in combating diabetes (Kam et al., 2016). Similarly, finger millet is also gaining popularity as an ideal target crop for Ca biofortification.

## Why use finger millet as a model for calcium biofortification?

Finger millet possesses all the quantitative and qualitative traits to serve as a model for Ca biofortification. It stands out as the richest source of Ca among all the cereals (Table 1). It has three times more Ca than milk and 10-fold higher Ca than brown rice, wheat or maize (Kumar et al., 2016a). Besides Ca, finger millet is also very rich source of iron, amino acids like methionine, slowly digestible starch and phytochemicals like polyphenols. It is a gluten-free, low fat cereal which is non-allergic and easily digestible. For these characteristics, it is often termed as a “super cereal” (Kumar et al., 2016a). Apart from its nutritional attributes, finger millet has excellent environmental sustainability credentials. It can easily withstand harsh climatic conditions, low soil fertility, requires very little inputs with a short growing season (Kumar et al., 2016a). It can reach the yield potential of up to 10 tons/ha under optimum irrigated conditions (Padulosi et al., 2015). It has excellent storage quality traits and can be valuable in areas where farmers suffer losses due to dearth of post-harvest management.

**Table 1:** Calcium content of various cereals.

**Cereal**	**Calcium content (mg/100 g edible portion)**	**References**
**MILLETS**		
Finger millet (*Eleusine coracana* L.)	344	Shobana et al., 2013
Teff (*Eragrostis teff*), mixed	78.8–147	Baye, 2014
Fonio (*Digitaria exilis*)	44	National Research Council, 1996
Pearl millet (*Pennisetum glaucum*)	42	Shobana et al., 2013
Foxtail millet (*Setaria italica*)	31	Shobana et al., 2013
Kodo millet (*Paspalum scrobiculatum*)	27	Shobana et al., 2013
Barnyard millet (*Echinochloa crus-galli*)	20	Shobana et al., 2013
Little millet (*Panicum sumatrense)*	17	Shobana et al., 2013
Proso millet (*Panicum miliaceum*)	14	Shobana et al., 2013
**COMMON CEREALS**		
Wheat (*Triticum aestivum*)	41	Shobana et al., 2013
Rice (*Oryza sativa*) brown	33	Saleh et al., 2013
Corn (*Zea mays*)	26	Saleh et al., 2013
Sorghum (*Sorghum bicolor*)	25	Saleh et al., 2013
Barley (*Hordeum vulgare*), raw	20	McKevith, 2004
Rye (*Secale cereale*), flour	20	McKevith, 2004
Oatmeal (*Avena sativa*), quick cook raw	52	McKevith, 2004
Rice (*Oryza sativa*) raw milled	10	Shobana et al., 2013

Therefore, integration of a naturally Ca-rich crop like finger millet in global biofortification programs can be a good starting point to alleviate Ca malnutrition (Sharma et al., 2017). Given that women share a significantly higher proportion of osteoporotic morbidity (“Facts and Statistics,” International Osteoporosis Foundation, http://www.iofbonehealth.org/facts-statistics), regular consumption of finger millet during and after pregnancy as well as lactation can provide significant benefits to maternal and child bone health. Another advantage of Ca-enriched finger millet over expensive commercially fortified foods is its affordability to these malnourished areas. For low-income households which mostly subsist on starchy and bulky foods like rice and cassava for their calorie requirements (https://www.devex.com/news/better-crops-for-better-nutrition-86583), finger millet ensures a pragmatic solution that no family member (especially women and children) suffers from Ca deficiency.

## Prospecting bioavailability of calcium from biofortified finger millet to achieve adequate intakes

Determining the efficacy and biological impact of Ca-biofortified finger millet on better nutrition and improved health is very challenging. It depends mainly on two processes: bioaccessibility and bioavailability of Ca in the seeds. Bioaccessibility is a measure of the nutrient fraction available for absorption after its release from food matrix in the gastrointestinal tract. On the other hand, bioavailability is a utilization-based definition, where the ingested, digested and absorbed nutrient reaches the systemic circulation and exerts a positive effect on health (Carbonell-Capella et al., 2014). Solubility, dialysability and gastrointestinal model are generally used as *in vitro* methods for measuring Ca bioaccessibility. On the other hand, evaluation of Ca bioavailability is ideally evaluated through *in vivo* human studies. However, considering the complexity of large-scale human trials and ethico-legal procedures, Caco-2 cell culture, which behave like human intestinal cells, can provide an alternative analysis.

It is well known that only <30% of the consumed Ca is effectively absorbed (Heaney, 2006). Interestingly, using the *in vitro* bioaccessibility methods, uncooked finger millet has been found to have 36.6% soluble and 28% dialyzable and bioavailable Ca (Amalraj and Pius, 2015). This is higher than rice (30.4% soluble Ca; 24.7% dialyzable Ca), sorghum (31.9% soluble Ca; 26.0% dialyzable Ca) and maize (25.4% dialyzable Ca). Therefore, finger millet in itself is an effective source of bioavailable Ca than many other staple cereals and its improvement through biofortification is an effective strategy that can relegate Ca deficiency.

However, the fate of actual Ca bioavailability from finger millet relies on and is challenged by several other factors. These include intrinsic grain property (solubility, interaction with other constituents of the food matrix) and extrinsic factors (condition of the host, food processing and storage). Therefore, a better understanding of how these factors influence and impact Ca efficacy becomes essential before the biofortified finger millet can carve a path in the farmer and consumer markets.

### Grain's intrinsic factors that impact Ca bioavailability in finger millet

Many plant-based Ca sources have limited accessibility of Ca for absorption due to the formation of insoluble complexes. Phytate and oxalate are two such bioavailability limiters that can impede Ca absorption as they exhibit a strong negative correlation with Ca bioaccessibility (Kamchan et al., 2004; Gibson et al., 2010; Krishnan et al., 2012). Several studies in legumes and cruciferous vegetables have reported that high *in vitro* Ca solubility and dialysability corresponds to low levels of phytate, oxalate and dietary fiber (Lucarini et al., 1999; Kamchan et al., 2004). In cereals, phytate and oxalate were shown to account for 7 and 15–20% inhibition of Ca bioavailability, respectively (Amalraj and Anitha Pius, 2015). In wheat and barley, phytate, but not fiber, has been proclaimed as having the major inhibitory effect on Ca absorption (Kennefick and Cashman, 2000). Phenolic compounds like tannins reduce the bioavailability of minerals by forming insoluble complexes with divalent metal ions (Rao and Prabavathi, 1982). An *in vivo* digestibility trial on birds fed on low (1%), medium (2%), and high (3%) tannin sorghum diets showed that as compared to control, the Ca absorption reduced by 1.22, 1.67 and 2.22 fold, respectively (Mahmood et al., 2014).

Likewise, finger millet also contains these antinutrients that negatively affect grain palatability and can be a constraint to its Ca bioaccessibility. There is a wide range of phytate and oxalate content in finger millet based on the genotypes. The phytate content in finger millet ranges from 679 mg/100 g to 1,419.4 mg/100 g (Antony and Chandra, 1999; Makokha et al., 2002). The grains have been found to contain higher phytate content (783.5 mg/100 g) than rice (289.9 mg/100 g), pearl millet (518.5 mg/100 g) and sorghum (571.1 mg/100 g) but lower than wheat (792.1 mg/100 g) and maize (851.5 mg/100 g) (Amalraj and Pius, 2015). Similarly, finger millet grains have been reported to contain oxalic acid to the extent of 45.7 mg/100 g (Rachic and Peters, 1977). Out of the total oxalate fraction present in the food matrix, soluble oxalate has the ability to bind Ca and reduce its absorption. In a recent study, it was found that finger millet has higher total oxalate content (11.3 mg/100 g) than other cereals (except pearl millet; 20.0 mg/100 g) but had the lowest percentage of soluble oxalate (45.9%) among other cereals (Amalraj and Pius, 2015). Even though the phytate and total oxalate content of finger millet are higher than many other cereals, it still contains more bioavailable Ca percentage (28%) than rice (24.7%), maize (25.4%), and sorghum (26%) (Amalraj and Pius, 2015).

Finger millet grains also have a wide range of total phenolics and tannins content (Devi et al., 2014). Tannin content estimation has revealed that the African varieties of finger millet have about three times more tannin percent than the Indian varieties (Ramachandra et al., 1977). Finger millet has been shown to contain up to 264.1 mg/100 g tannin (Amalraj and Pius, 2015). This is much higher than maize (25.5 mg/100 g) and rice (14.3 mg/100 g) but lower than pearl millet (275.8 mg/100 g) and wheat (287.3 mg/100 g) (Amalraj and Pius, 2015). Despite the knowledge of the extent of varietal variations in tannin content of finger millet, their direct effect on inhibition of Ca absorption and bioavailability has yet not been investigated.

Calcium absorption may also be affected by the non-digestible oligosaccharides and dietary fibers in the food matrix. These compounds reduce the activity of digestive enzymes and slow down the digestion process. Dietary fibers were found to be significantly associated to Ca bioavailability in commercially available rice flakes (Suma et al., 2007). The indigestible starch component (resistant starch) has been found to increase Ca absorption in rats, probably by enhancing its solubility (Schulz et al., 1993). Finger millet is also a great source of resistant starch (Devi et al., 2014). It also has the highest total, soluble and insoluble dietary fiber when compared with wheat, rice, maize, sorghum and pearl millet (Amalraj and Pius, 2015). The role of such factors present in finger millet and other grain crops is generally considered positive in Ca absorption but the magnitude of their stimulatory effect requires further validation by *in vitro* or *in vivo* methods.

For enhanced Ca bioavailability from finger millet, grain Ca content needs to be improved with a concomitant but conscious effort for the reduction of antinutrient compounds. This is because these compounds play a vital role in plant development and survival. For example, finger millet tannins are effective in reducing pre- and post-harvest losses as they provide protection against molds, insects and other abiotic stress (Gull et al., 2014). Similarly, phytic acid acts as the main phosphorus store for the seeds (Singh and Raghuvanshi, 2012). These compounds have also called attention due to their nutraceutical value and protective effects against many chronic diseases (Kumar et al., 2016a). Thus, their importance can never be completely disregarded. Engineering their content to become a non-limiting factor in Ca absorption from finger millet must be done in a way that does not negatively affect crop performance. A justified way to accomplish this is by employing efficient and suitable grain processing techniques.

### Influence of grain processing on Ca bioavailability

Processing techniques of the grain can affect the total mineral content and factors associated with their bioaccessibility. Finger millet is a very versatile cereal and can be processed and utilized in numerous ways while retaining the available Ca. Popping by high temperature and short time (HTST) treatment was found to have no effects on the total Ca content in finger millet but lowered the Ca bioaccessibility and polyphenol content by as much as 19 and 22%, respectively (Krishnan et al., 2012). Microwave cooking by boiling also does not greatly improve the percentage of soluble or bioavailable Ca (Amalraj and Pius, 2015). This implies that HTST-based processing and microwave cooking methods do not favor improved bioaccessible Ca from finger millet.

On the other hand, sprouting of finger millet improves the extractability of Ca and lowers antinutrients like phytate and tannins to an undetectable level after 4 days (Mbithi-Mwikya et al., 2000). Flour made from whole grain finger millet was found to have higher Ca content (325 mg/100 g) than those made from decorticated (222 mg/100 g) one (Hemanalini et al., 1980). Decortication is a process of removal of the seed coat matter which is responsible for lowering Ca bioaccessibility. It is interesting to note that processing by decortication significantly improved Ca bioavailability in rats and this was attributed to its low fiber and phytic acid content (Hemanalini et al., 1980). In an evaluation of various processing methods of finger millet on Ca bioaccessibility, seed decortication and malting were found to be the most efficient techniques (Krishnan et al., 2012). Decortication improves Ca bioaccessibility by 37.5%, in spite of lowering the total Ca content by 40%. This increase was attributed to a direct decrease in inhibitory contents present in the seed coat like phytic acid (31% reduction) and polyphenols (70% reduction). Malting, which involves germination and thermal treatment, also influences the bioaccessible Ca content of finger millet in a positive way (Platel et al., 2010; Krishnan et al., 2012). Again, this is because germination process greatly reduces the concentration of phytic acid and polyphenols. Fermented flour or sprouting followed by fermentation also showed marked enhancement in Ca availability (20%) with a concomitant decline in phytates, phenols, tannins, and trypsin inhibitor activity (Sripriya et al., 1997; Antony and Chandra, 1998; Makokha et al., 2002).

The above examples establish beyond doubt that bioaccessibility of Ca from finger millet can be even further improved by simple processing methods which can be scaled up to industrial levels. These methods have potential to add values to both traditional as well as to contemporary value-added food products improving their edible and sensory properties (Hotz and Gibson, 2007; Shobana et al., 2013; Verma and Patel, 2013).

### Host (extrinsic) factors that influence Ca bioavailability

Apart from the Ca bioavailability parameters, the capability to determine the effect on Ca status on target populations is another specific challenge. Host factors, such as age, gender, dietary patterns may show differential effects of finger millet-based diets on the net Ca contribution. These factors must be considered in controlled feeding community-based studies to determining the biological impact of biofortified crops.

In the past, various attempts have been put together to assess the Ca bioavailability *in vivo*. Early nutrition studies have shown that rats fed with a diet composed of 70% finger millet retain 68% Ca (Giri, 1940). A further reduction of the finger millet content to 20–40% in diets contributed to increased Ca retention to 84–88% levels (Giri, 1940). This shows that even a low dietary component of finger millet is sufficient to maintain Ca availability because of its high Ca content. In fact, in a more recent study, Ca from finger millet had shown to have a better uptake as compared to commercial Ca supplementation tablet (Bhide et al., 2013). In this *in vivo* study, the serum Ca level of rats fed with finger millet extract and a finger millet based ready-to-drink formulation was >35% higher as compared to conventional Ca tablet supplemented group.

However, for human metabolism studies, host factors like age and gender are important parameters to estimate daily requirement, intake and retention of dietary Ca. Many nutrition reports have estimated the contribution of finger millet for Ca homeostasis in humans. A study by Subrahmanyan et al. (1955) had found that a finger millet variety, H22, with Ca content 440 mg/100 g can on an average provide 3.4 g Ca/ day to healthy adult males aged 22–32 years. This amounted to Ca retention of 98 mg (approximately 3%) from a total daily intake of 3.4 g/day. This was higher than a brown bread or Ca carbonate fortified brown bread diet providing only 0.5–1.2 g Ca/day, respectively (McCance and Widdowson, 1942). It is recommended that diets should provide at least 200 mg/100 g of Ca to counteract the anticalcifying effect of phytic acid (McCance and Widdowson, 1942). Interestingly, 86% of phytate ingested from the finger-millet-based diet was hydrolysed during digestion and absorption process (Subrahmanyan et al., 1955). As most of the phytate is broken down during digestion, therefore, regular inclusion of finger millet in diet can efficiently maintain a positive Ca balance. In another study, young girls (aged 9–10) were fed on four different diets containing only rice, 75% rice + 25% finger millet, 50% rice + 50% finger millet and only finger millet as the primary cereal (Joseph et al., 1959). About 19% Ca was retained from just rice-based diets which increased to 22.5–25.3% when a portion of rice was replaced with 25–50% finger millet. Therefore, finger millet can naturally contribute to boost the Ca status across ages evidently surpassing the other cereals. One serving of finger millet-based Ca-rich products, which were processed to increase Ca bioavailability, was shown to provide >0.2 g Ca contributing to 25% of Indian RDA of Ca for children and adolescents (Sanwalka et al., 2011). It is interesting to note that the Ca retention capacity of children seems to be much higher than adult subjects. This may be because growing age lowers digestibility and retention capacity of Ca and hydrolysis of antinutrients like phytate (Yoshida et al., 1983). It needs to be stressed here that most of these studies have been conducted more than 50 years ago. However, lifestyle and dietary patterns have drastically changed in recent years. Therefore, evaluating positive Ca impacts of finger millet diet across various groups needs to be measured keeping in view the current scenarios as well.

In a case study in rural India, the dietary patterns of women self-help groups was assessed for their nutrient adequacy (Vijayalakshmi et al., 2010). The families usually consumed one portion of finger millet preparation two times a day along with one portion of pulses and vegetables. It was found that irrespective of the socio-demographic profile of the subjects (like age, education, family income, family size), Ca levels met the recommended RDA and their adequacy was attributed to regular finger millet inclusive diet. As no major replacement of diet is necessary, finger millet and its derived food products have the advantage to be more acceptable to the people. This makes it a more viable option to be effective providing adequate Ca intakes and prevent Ca deficiency. These reports provide an idea that designing food products from biofortified varieties of finger millet can easily supplement and add-on to the daily Ca intake across ages and genders with various dietary practices. Such information can allow the acceleration of finger millet biofortification programs.

## Major challenges to develop finger millet as a model for calcium biofortification

In order to develop Ca biofortified finger millet, nutritionists must have available resources (superior Ca-rich varieties), a well-developed methodology to evaluate the bioaccessibility (*in vitro* and *in vivo*), awareness of the limiting factors (enhancers and inhibitors), and prior assessment of efficacy and effectiveness of Ca biofortified food. However, currently the development in this area is extremely limited and developing more nutritious varieties is challenging. Given that millet biofortification has recently been strategized, development of research programs for Ca-biofortified finger millet will have to address some arguments as discussed below.

### Challenges to efficiently utilize the available germplasm resources and genetic diversity

In recent years, various efforts have been made by geneticists and breeders to identify naturally occurring genetic diversity in finger millet. However, the major challenge at present is how these resources could be exploited to develop Ca-biofortified finger millet. Currently, finger millet genebanks across the globe conserve more than 37,000 accessions with India, Kenya, Ethiopia, Uganda, and Zambia housing the major collections (Vetriventhan et al., 2015). As of now, the entire genetic diversity present among the finger millet germplasm is available as small sets (core) and sub-sets (mini core) collections (Vetriventhan et al., 2015). Using these collections, 15 accessions were identified as most promising (3.86–4.89 g/kg) for further improving grain Ca content in cultivated finger millet (Upadhyaya et al., 2011). Recently, another core set of 77 germplasm of Indian and African origin has been formed using the base germplasm of finger millet 1,000 accessions (Chandrashekhar et al., 2012). In addition, finger millet composite collections (1,000 accessions) and a derived reference set (300 accessions) representing region- and race-based available diversity of the entire collection, is also available (Upadhyaya et al., 2005; Upadhyaya, 2008). Although, these large collections of finger millet germplasm serve as an ideal resource to be utilized in improving its Ca concentration, a majority of it remains largely underutilized for breeding high Ca finger millet varieties. Some of the main reasons for this lag are due to factors, such as weak and insufficient strategies for harnessing the useful genetic diversity available in these collections, barriers related to introduction and crossing of exotic germplasm, few pre-breeding programs to facilitate introgression of desirable nutrition quality into breeding lines and re-circulation of same working collections by breeders (Dwivedi et al., 2009; Upadhyaya et al., 2014). Although such precious germplasm collections exist, sometimes there are also practical barriers associated with their availability for use. For example, restricted global exchange of accessions due to legal aspects of seed transfer agreements poses a limitation for verification of adaptability to multi-location or multi-environment trials (Nass et al., 2012). Even a dearth of trained finger millet breeders to meet the demand can be an issue for making use of this treasure.

In addition, a huge range of diversity for grain Ca also exists within the gene pool of finger millet which remains to be exploited in targeted breeding. Two main gene pools exist for this species, namely *Eleusine coracana* sub-species *africana* (wild progenitor) and *E. coracana* sub-species *coracana* (domesticated cultivars/varieties and landraces (Agarwal and Maheshwari, 2016). Considerable variation in grain Ca contents has been observed in both the genepools. A detailed systematic analysis of grain Ca content in *E. coracana* sub species *africana*, from Ethiopia, revealed significantly higher Ca (515 mg/100 g) than the domesticated *E. coracana* sub species *coracana* from Kenya (401 mg/100 g) and India (375 mg/100 g) (Barbeau and Hilu, 1993). Many pieces of evidence for genotype effect in finger millet Ca content also exist. Vadivoo et al. (1998) found a large heritable genetic variation in relation to Ca content in 36 genotypes of finger millet, with white seeded varieties containing moderate levels of Ca. Based on their results, Malawi 1915 (486.7 mg/100 g Ca) and CO 11 (487 mg/100 g Ca) genotypes were proposed to be employed as crossing parents in breeding for the improvement of Ca content. Similarly, the dark red to very dusty red colored finger millet genotypes, CO 10, KM 1 and MI 302 sourced from the Dry zone Agricultural Research Station, Sri Lanka were shown to contain 240–250 mg% Ca (Ravindran, 1991). In another report, the white seeded finger millet varieties showed higher average Ca (329 mg%) than the brown seeded ones (296 mg%) (Seetharam, 2001). In spite of the extensive screening of finger millet germplasm for grain Ca content, the identified potential candidate genotypes have remained unused for developing higher Ca containing varieties breeding.

At the same time, just selecting suitable donor lines for selective breeding based on variation in grain Ca content is not sufficient and may not even be successful as such variations may often be regulated at various other levels. Therefore, determination of genetic stability and adaptability of this trait across multiple environments is one of the prerequisite to develop effective strategies for breeding elite lines. However, a severe gap exists in our knowledge about accuracy by which genetic variation for Ca content can be reproduced by finger millet genotypes grown across various agro-ecological conditions.

### Challenges associated with breeding-based genetic improvement of finger millet

As finger millet is a naturally self-pollinating crop, artificial hybridisation by crossing of suitable parental lines is often a difficult task. Mass and pure-line selection practices have come in handy for inter-varietal improvement for grain yield, early maturity and disease resistance (Harinarayana, 1986; Table 2). For example, using pure line selection from the germplasm accession, finger millet culture WWN-25 has been released as a high yielding variety, GNN-7, for cultivation in Gujrat state of India (Patil et al., 2016). This is a promising development as this variety contains higher Ca (468.0 mg/100 g) than the national check variety VR-708 (398.0 mg/100 g) without compromising on the yield. However, optimum deployment of other breeding methods, such as recombination breeding, for generating stable hybrids, breeding progeny and inbred lines has been delayed due to challenging biparental cross, difficult emasculation and artificial hybridization in finger millet. To overcome these challenges, induced mutations, such as genetic male sterile systems (viz., INFM 95001 reported by ICRISAT; http://oar.icrisat.org/618/1/PMD_71.pdf) have proved to be another efficient breeding tool for yield and disease resistance in finger millet. These systems and their subsequent breeding can be used effectively to increase the genetic variance by creating new recombinants and segregating populations by exploiting the genetic background. Therefore, developing genetic resources for finger millet, such as mapping populations, breeding lines and male-sterile mutant lines (Gupta et al., 1997; Krishnappa et al., 2009; Parashuram et al., 2011), deserves attention. Such material will be immensely valuable for tagging nutritional quality traits, especially grain Ca content, and thus facilitate genetic biofortification of finger millet.

**Table 2:** Modern finger millet varieties released in the last decade.

**Variety**	**Agro ecology/region where best suited**	**Country of release**	**Release Year**	**Maturity period (days)**	**Grain yield (t/ha)**	**Special attributes**	**References**
Okahale-1 (KAK-WIMBI 1)	Low-high altitude [0–2,500 meter above sea level (masl)]	Kenya	2016	88–138	1.1–6.7	High yielding, large panicles, resistant to blast and striga, non-lodging, drought tolerant, brown grain color, robust plant type	http://www.kephis.org/images/VarietyList/april20170525.pdf
I.E. 4115 (KAK-WIMBI 2)	Low-high altitude (0–2,500 masl)	Kenya	2016	88–131	1.3–6.0	Resistant to blast, striga, and lodging, drought tolerant, brown grain color	
KACIMMI 42 (KAK-WIMBI 3)	Low-high altitude (0–2,500 masl)	Kenya	2016	87–130	1.3–6.4	Resistant to blast, striga, and lodging, drought tolerant, brown grain color	
Maseno 60D	Lowlands-mid altitudes (0–1,500 masl)	Kenya	2016	80–90	3.1	Extra early, resistant to drought	
U-15 (Maridadi)	Low-medium altitude (1–500 masl); Western Kenya, Nyanza, Eastern counties, Rift Valley, all Coastal counties	Kenya, Uganda, Tanzania	2011	90–120	1.1–4.9	Early maturity, high yield, brown grain color, resistance to lodging, blast and striga, drought tolerant	
P 224	Medium-high altitude 1,150–1,750 masl	Kenya, Uganda, Tanzania	–	90–120	2.2–5.0	Tall, tolerant to lodging and blast disease, easy to thresh, light colored grain	
KAT/FM-1 (Katumani Finger Millet-1)	Low-high altitude (50-2,000 masl)	Kenya, Uganda, Tanzania	–	90–115	1.0	Drought and blast tolerant, resistant to lodging, high in calcium	http://www.kalro.org/alris/index.php/home/print_variety/20160927113240
Gulu-E	–	Uganda, Kenya	–	110–115	2.2	Early maturity, high yield, drought escaping, tolerant to blast disease, easy to thresh	http://www.kalro.org/fileadmin/publications/brochuresI/Fingermillet.pdf
KMR 340	Karnataka	India	2016	90–95	35–40	White colored, suitable for confectionary, resistant to blast and blight diseases, tolerant to stem borer and aphids	http://millets.res.in/technologies/finger_millets_varieties.pdf
VL Mandua 376	All finger millet growing areas	India	2016	103–109	3.2–3.4	Responsive to fertilizer and moderately resistant to blast	
VL Mandua 348	Uttarakhand	India	2016	104–112	18–20	Resistant to neck and finger blast, non-lodging, light copper colored grains, suitable for organic cultivation	
GNN-7 (Gujarat Navsari Nagli-7)	Kharif; rainfed crop in south and middle Gujarat	India	2016	125	2.7	White colored grains, medium duration, high yield, easy threshability, non-lodging, moderate resistance to blast, calcium: 468 mg/100 g	Patil et al., 2016
VL Mandua 352	All finger millet growing states except Tamil Nadu and Maharashtra	India	2014	95–100	3.6–3.9	Early maturing, moderately resistant to blast, high yielding	Gupta et al., 2015
VL Mandua 347	All finger millet growing states, especially mid and higher hills	India	2012	95–100	2.2–2.4	Early maturing, moderately resistant to blast, rich in iron (7.9–8.0 mg/100 g) and zinc (3.56–3.7 mg/100 g)	Gupta et al., 2013
INDIRA RAGI-1	Suitable for early monsoon conditions in Chattisgarh	India	2012	120–125	2.8–2.9	Semi dwarf, late maturity, moderately resistant to neck and finger blast and tolerant to stem borer, Non shattering, non-lodging and responsive to fertilizers, suitable for early monsoon condition, can withstand water stress	http://www.dhan.org/smallmillets/docs/report/Compendium_of_Released_Varieties_in_Small_millets.pdf
PPR 2700 (VAKULA)	Andhra Pradesh region	India	2012	105–110	2.8–3.3	Semi dwarf plants, Medium duration, Resistant to leaf blast and drought tolerant	
VR 936 (HIMA)	Andhra Pradesh	India	2012	115–120	3.1–3.3	Suitable for late conditions, dull white grains, responsive to nitrogenous fertilizers	
KMR 204	Karnataka	India	2012	100–105	3.3–3.9	Early duration and high yielding	
OEB 532	Odisha, Bihar, Chattisgarh, Karnataka, Tamil Nadu	India	2012	110–115	2.4–2.8	Moderately resistant to blast diseases, non- lodging and non-shattering, highly tolerant to myllocerus weevil, earhead caterpillars, stem borer and grass hopper	
OEB 526	Odisha, Bihar, Chattisgarh, Karnataka, Tamilnadu	India	2011	110–115	2.8–2.9	Moderately resistant to leaf, neck and finger blast diseases, light brown colored grains	
KOPN 235 (PHULE NACHNI)	Sub mountain and western ghat zones of Maharashtra	India	2011	115–120	2.8–2.9	Semi dwarf, late maturity, resistant to blast	
KMR 301 (GOWRI)	Southern dry zone of Karnataka, rabi season	India	2009	120–125	6.1–6.6 (Irrigated) 3.9–4.4 (Rainfed)	High grain and straw yield, tolerant to blast	
VR 847 (Srichaitanya)	Andhra Pradesh	India	2009	110–115	2.9–3.1	Moderately resistant to blast, tolerant to caterpillars and aphids	
GN-5 (Gujarat Nagli-5)	Hilly area of south Gujarat and middle Gujarat region	India	2009	120–130	3.4	White colored grains, high yielding, easy to thresh, non-lodging, moderately resistant to leaf blast, resistant to neck and finger blast, non-lodging, calcium: 432 mg/100 g	Chaudhari et al., 2012
GPU 67	Widely adapted across Chattisgarh, Jharkhand, Karnataka, Maharashtra, Tamil Nadu and Uttarakhand	India	2009	115–120	4.4–5.0	Semi dwarf, moderately resistant to neck and finger blast, calcium: 219 mg/100 g, profuse tillering, copper brown seeds	http://www.dhan.org/smallmillets/docs/report/Compendium_of_Released_Varieties_in_Small_millets.pdf http://www.dhan.org/smallmillets/docs/report/Compendium_of_Released_Varieties_in_Small_millets.pdf http://www.dhan.org/smallmillets/docs/report/Compendium_of_Released_Varieties_in_Small_millets.pdf http://www.dhan.org/smallmillets/docs/report/Compendium_of_Released_Varieties_in_Small_millets.pdf
GPU 66	Late kharif season in Karnataka	India	2009	112–115	3.9–4.4	Resistant to neck and finger blast	
VR 762 (Bharathi)	Andhra Pradesh	India	2006	110–115	3.1–3.3	Moderately resistant to blast, reddish brown colored grains, tolerant to ear caterpillars and aphids	
PRM 1	Hilly regions of Uttarakhand	India	2006	110–115	2.2–2.8	Resistant to blast, light copper colored grains	
Sailung Kodo-1	High hills (1,300–2,200 masl)	Nepal	2015	155	2.7	Non-lodging, moderately resistant to finger blast, neck blast and *Cercospora* leaf spot, calcium: 388 mg/100 g	https://cgspace.cgiar.org/bitstream/handle/10568/80892/Release_Joshi_2017.pdf?sequence=1
Kabre Kodo-2	Mid and high hills (700–1,800 masl)	Nepal	2015	153	2.8	Drought tolerant, non-lodging, field resistant to finger balst, neck blast and *Cercospora* leaf spot, calcium: 379 mg/100 g	

### Underutilization of available genetic and genomic resources for molecular breeding applications

Efforts have been made to generate molecular markers for characterizing important traits like grain Ca and protein content and resistance to blast infection in finger millet. Genomic tools like SSR markers have helped to assess the range of genetic diversity for grain Ca content in various finger millet genotypes (Panwar et al., 2010; Nirgude et al., 2014; Yadav et al., 2014; Kumar et al., 2015a). Nine SSR markers derived from candidate Ca transporter and sensor genes have been found to be significantly associated with the Ca trait. They could serve as an important functional resource for the genetic improvement of finger millet's nutritional value through marker-assisted breeding (MAB; Kumar et al., 2015a; Sharma et al., 2017). However, the inbreeding nature, limited recombination rates and a historical genetic bottle-neck during isolated domestication of this crop significantly impacts the extent of available genetic diversity in finger millet. Such loss of genetic diversity is a challenge for geneticists and breeders working with a limited number of finger millet accessions. Further, until recently, there has been no progress in application of the finger millet genetic map in trait mapping despite the assembly of the only molecular marker-based linkage map a decade ago (Dida et al., 2007; Srinivasachary et al., 2007). It still remains under-utilized for tagging and identification of genes/quantitative trait locus (QTL) governing grain Ca content probably due to an insufficient number of informative markers.

The unavailability of sufficient markers and genome sequence information in finger millet has resulted in limited breeding efforts for nutritional improvement. Nonetheless, advances in large-scale genomics technology have now streamlined production of genome-wide markers which can be used for large-scale identification of candidate genomic loci. This advancement has also been capitalized to generate single nucleotide polymorphism (SNP) markers in finger millet using genotyping-by-sequencing (GBS; Kumar et al., 2016b) and Roche 454 and Illumina sequencing (Gimode et al., 2016). Inspite of the low level of polymorphisms in cultivated finger millet genotypes (Salimath et al., 1995), these SNPs may provide some explanation for variation in grain Ca content among finger millet genotypes. However, before their utilization, it is crucial to differentiate true SNPs among different genotypes from the homeologous SNPs within an individual genotype due to allotetraploidy (AA and BB sub-genomes) of finger millet.

Advances in cereal genomics have elaborated that genomic level similarities are conserved in the relative physical positions across species both on a fine scale (co-linearity) as well as on a chromosomal scale (synteny). For example, comparative mapping of finger millet to rice has shown a fairly high level of collinearity among these crops (Srinivasachary et al., 2007). The availability sequence information for several members of the grass family is now assisting in the development of inter-species molecular markers and gene discovery in unexplored crop genomes, such as finger millet (Wang et al., 2005; Kalyana Babu et al., 2014a,b). Until finger millet whole genome sequence becomes available, such comparative genomics approaches can benefit finger millet improvement programs. However, identification of common quantitative trait loci (QTLs) that control grain Ca content among cereals remains to be explored. This will further facilitate identification of orthologous regions and transfer of genetic information across species for improving Ca content of other millets and non-millets.

### Inadequate understanding of Ca homeostasis mechanisms in finger millet

Emphasizing on the molecular status of Ca accumulation in finger millet grains, various genes have recently been identified (Sood et al., 2016). More recently, a molecular model for Ca transport from soil to seed has been proposed (Sharma et al., 2017). This hypothetical model is based upon genes that are differentially expressed in contrasting finger millet cultivars (Mirza et al., 2014) or grain filling and developing spike transcriptome studies in this crop (Singh et al., 2014, 2015; Kumar et al., 2015b). These genes correspond to Ca sensing and binding, Ca transport and seed storage, such as, *type IIB ATPase, Ca*^2+^*/H*^+^ *antiporter* (*CAX1*), two *pore channel* (*TPC1*), calmodulin (*CaM*), CaM-dependent protein kinases (*CaMK1* and *CaMK2*) and *14-3-3* (Table 3). High Ca accumulation in finger millet has been mainly attributed to the Ca sensor genes which have been proposed as candidates for targeted Ca enhancement in finger millet varieties (Singha et al., 2016). Alteration in the genes expression levels only indicates a pattern among the contrasting genotypes which does not always validate the translated protein products involved in Ca accumulation. A few recent studies have characterized accumulation of Ca-binding protein (calreticulin) and CaM protein during grain filling stages of finger millet (Kumar et al., 2014; Singh et al., 2016). Unfortunately, large-scale protein profiling to identify the complete set of proteins involved in finger millet Ca homeostasis is still unavailable.

**Table 3:** List of important genes identified for calcium uptake, transport and storage in finger millet.

**Gene/Transcript**	**Tissue**	**Plant**	**Method**	**References**
**CALCIUM SENSORS**				
EcCaML8, EcCaML11, EcCaML14, EcCaML18, EcCaML29, EcCaML35, EcCaML38	Developing Spike	Genotypes GP-1 (low Ca content) and GP-45 (high Ca content)	Transcriptome analysis, differential expression	Singh et al., 2014
EcCBL2, EcCBL4, EcCBL6, EcCBL9, EcCBL10				
EcCIPK5, EcCIPK9, EcCIPK11, EcCIPK19, EcCIPK24, EcCIPK29, EcCIPK31				
EcCRK2, EcCRK3				
EcCDPK4, EcCDPK13, EcCDPK17				
**CALCIUM UPTAKE AND TRANSLOCATION**				
CAX1, TPC1, Ca^2+^ ATPase, CaMK1	Roots	Genotypes GP-1 (low Ca content) and GP-45 (high Ca content)	Differential expression	Mirza et al., 2014
CAX1, Ca^2+^ ATPase, CaMK1, CaMK2	Stem			
CAX1, 14-3-3, CAM	Leaves			
CAX1, Ca^2+^ ATPase, CAM, CaMK2	Spike: inflorescence immergence			
CAM	Spike: anthesis			
CAX1, CAM, CaMK2	Spike: grain filling			
CAX1, TPC1, CAM, CaMK2, 14-3-3	Spike: grain maturation			
**CALCIUM TRANSPORTERS**				
EcCAX4, EcVCAX1a, EcVCX2, EcCAX1b, EcCAX3, EcNaCa11exA, EcNaCa11exB	Developing Spike	Genotypes GP-1 (low Ca content) and GP-45 (high Ca content)	Transcriptome analysis, differential expression	Singh et al., 2015
EcPM8ATPase, EcPM4ATPase8, EcPM12ATPase, EcPM2ATPase9, EcPM1ATPaseII, EcPM12ATPase4, EcPM5ATPase4, EcPM3ATPaseB, EcPM3ATPase2, EcER3ATPase4, EcER3ATPase3				
EcCL1 pore				

Even though the past and current developments have generated a huge wealth of transcriptomic datasets linked with the Ca uptake, translocation and accumulation in finger millet, without functional characterisation, these candidate genes are merely speculation. With a lack of evidence for efforts being made to functionally characterize these genes/proteins, it is possible that these potential candidates may not have expected impact on enhancing grain Ca content. While, it is practically impossible to functionally characterize all genes linked with Ca acquisition, sensing and accumulation, transgenic technology along with progress in gene editing (such as CRISPR-Cas and TALEN) can aid in a better functional understanding of many candidate genes. Such advancements in gene editing technology along with well-established genetic transformation protocols for generation of transgenic finger millet (Kumar et al., 2016a) can significantly improve our understanding of this process.

Besides this, the role of other factors (for example, hormones, root morphological traits, endophytes, soil fertility, evapo-transpiration pull, translocation distance of Ca) on Ca homeostasis in finger millet remains unknown. This is a crucial question as the soil-plant interactions substantially affect the available proportion of micronutrient to the roots. In this context, soil medium supplemented with growth promoting rhizobacterium (*Azotobacter, Azospirillium*, phosphorus solubilizing bacteria) and vesicular-arbuscular mycorrhizal (VAM) fungi are generally practiced to enhance the grain yield and growth in finger millet (Ramakrishnan and Bhuvaneswari, 2014; Thilakarathna and Raizada, 2015). VAM are also known to increase the content and uptake of minerals, such as phosphate, nitrogen, zinc and copper (Tewari et al., 1993). The nutrient supply to the plant can be augmented either by efficiently mobilizing nutrient forms available in the soil or by extending the nutrient absorption surface by designing better roots system. However, how these associations impact Ca absorption and uptake in finger millet has received little experimental consideration. Further, the relationship between growth environments and climates may also alter xylem water flow, thus indirectly determining Ca distribution. So far, the established mechanisms of Ca accumulation in finger millet have been developed on the basis of controlled growth conditions. However, under field conditions, the unique soil-root interaction can influence the Ca-sensing and transport differently. In addition, any confounding effects of agronomic traits, such as vegetative growth, yield, stress resilience and disease resistance on grain Ca accumulation are not clearly established. Therefore, to develop finger millet as a model for Ca biofortification, we need a comprehensive understanding of the mechanisms and other factors that may influence this trait.

### Potential of next generation sequencing (NGS) for improved finger millet varieties

Enormous progress has been made in the genomics technology through application of high-throughput, economical and quicker next generation sequencing (NGS) platforms. Extending the benefits of NGS to finger millet, a recent effort of *de novo* sequencing has allowed whole genome sequence assembly covering approximately 82% of total estimated genome size (Hittalmani et al., 2017). Evidence of higher collinearity with foxtail millet and rice as compared to other *Poaceae* species, and the available genome sequencing information may help allele discovery and candidate gene identification for agronomically important traits (Hittalmani et al., 2017) leading to faster development of improved varieties. In addition, GBS, which is a NGS platform-based highly multiplexed genotyping system, has also been applied for SNP generation (Kumar et al., 2016b). Thus, now it is feasible to generate a higher density of markers by genotyping core collections of finger millet thereby increasing the level of genetic diversity explored. This is crucial for a predominantly self-fertilized crop like finger millet because it is expected to have low recombination rates and high linkage disequilibrium (LD) which would otherwise narrow the genetic diversity. Thus, the more genetically diverse populations in finger millet core collections, together with the huge amount of relevant marker information generated through NGS platforms can directly contribute to improved mapping resolution of traits, such as Ca content through genome-wide association studies (GWAS). By statistically reconnecting variation in grain Ca content back to its underlying genetic polymorphism, it is possible to identify functional common variants in LD and genomic regions where major-effect genes and QTLs (that serve as the targets of marker-assisted selection) are located. The GWAS studies can confirm previously identified genes involved in Ca homeostasis mechanisms as well as spot putative novel candidates. However, the efficiency of GWAS depends upon accurate grain Ca content phenotyping data over multi-location/multi-year trials.

An extension of MAS, genomic selection (GS) is an upcoming methodology in the area of genomics-assisted breeding (Meuwissen et al., 2001). In this approach, genome-wide marker genotype data along with available phenotypic data for a tested (reference/training) population are used to predict the performance of an untested (breeding) population based on genomics estimated breeding values (GEBV). Thus, instead of identifying few large-effect loci associated with Ca content, the GEBV model can more accurately predict the expected phenotype of a broader breeding population. This significantly reduces the time and costs associated with phenotyping a trait like grain Ca content. Finger millet enjoys the availability of germplasm resources, such as the core collections, which can be utilized as test populations to build genomic prediction models. As GS eliminates the need for previous identification of major QTLs and their use in selection, it can substantially speed up the genetic gain in this “orphan” crop. Another advantage is that if GEBVs are efficiently evaluated, finger millet breeders can make appropriate selection choices even earlier in the program, thereby significantly saving time on the generation cycle (Heffner et al., 2010). However, the applicability of GS in finger millet and selection of superior genotypes are dependent upon precise measurement and heritability of Ca content, sufficient marker density, the extent of LD decay, effective design of training population and its genetic relationship with the breeding population (Varshney et al., 2014). Nevertheless, NGS can contribute in exploring the depth and breadth of genetic diversity across germplasm sets bringing forward a huge wealth of genetic information. This will eventually lead to a new horizon for finger millet Ca biofortification.

## Future prospects and conclusions

By virtue of its health benefitting properties and environmental sustainability, a traditional but less popular crop like finger millet offers excellent opportunities for biofortification breeding. A foremost priority from geneticists and breeders viewpoint is capturing and utilizing genetic diversity for Ca content in the elite finger millet gene pools (for example, by bringing new sources of variation through rare and unique alleles). For trapping such useful variations, advances in the next generation sequencing technology must be utilized in generating sufficient number of markers for characterizing marker-trait associations and genomics-assisted breeding. With the implementation of such high-throughput approaches, it will be much easier to investigate the genetic architecture of this trait through comparative mapping in other millets and non-millet species. Mining of markers tightly linked to other traits governing grain Ca content and discovery of underlying genes can be an alternate strategy to develop high Ca finger millet varieties through traditional or modern breeding approaches and transformation-based methods. For example, rather than just arbitrarily increasing grain Ca content, future direction should target improvement in efficiency to mobilize, acquire, transport and store Ca in more bioavailable forms in the edible portions.

From the health perspective, at this point, we almost completely lack the understanding of interplay among grain Ca, other micronutrients and antinutrients metabolism in human body. Any potential risks of reducing the antinutrient content in the grains should be evaluated with a view of their long-term effects on human health. Therefore, the focus should be on demonstrating finger millet's bio-efficacy in order to monitor any potential negative trade-offs and unintended effects. In addition, experimental confirmation of *in vivo* Ca bioavailability needs to be cautiously attempted through designing pilot feeding studies for vulnerable groups (like children, nursing or post-menopausal women). Although the biological effects of improved finger millet may be relatively modest, it will have potential benefits in improving healthcare cost savings by reducing the risk of osteoporotic fractures and DALYs lost.

While agronomic factors can influence acceptability of the improved finger millet varieties by farmers, other parameters are important from the view of consumer acceptance. The extent of Ca recommended dietary allowance proportion as well as the sensory satisfaction supported by finger millet is essential in terms of developing food products keeping in line with recent lifestyle changes. Even after successful Ca biofortification of finger millet, its introduction and success as a functional food still entails knowledge of adequate food processing strategies (to minimize the nutrient loss) and study of consumer preferences. Further, communication support and the creation of market demand for its value-added products will be necessary. Therefore, a multi-disciplinary research approach, incorporating nutrition, health, agriculture, along with policy and market research, is needed to ensure the impact of high Ca-biofortified finger millet. Overall, it is worthwhile to conclude that finger millet biofortification will improve the quality of life for both the rural subsistence farming families as well as the consumers.

## Author contributions

SP and RY conceptualized the manuscript. SP wrote the manuscript. JK, PS, HO, and RY assisted and edited the manuscript. SP, JK, PS, and RS contributed in critically revising the draft and updating the manuscript for publication.

### Conflict of interest statement

The authors declare that the research was conducted in the absence of any commercial or financial relationships that could be construed as a potential conflict of interest.
